# Endogenous cathelicidin protects against *Toxoplasma gondii-*associated liver damage

**DOI:** 10.1128/iai.00708-25

**Published:** 2026-02-23

**Authors:** Yi Lin Tan, Paloma Cavalcante, Karina M. Cirone, Jacquelyn Tran, Franco Fiorani, Daniel Young, Antoine Dufour, Constance Finney, Eduardo R. Cobo

**Affiliations:** 1 Faculty of Veterinary Medicine, University of Calgary 70404, Calgary, Alberta, Canada; 2 Faculty of Agricultural Sciences, National University of Mar del Plata 683330 , Balcarce, Buenos Aires, Argentina; 3 McCaig Institute for Bone and Joint Health, Snyder Institute for Chronic Diseases, Hotchkiss Brain Institute, Cumming School of Medicine, University of Calgary 157742, Calgary, Alberta, Canada; 4 Department of Physiology & Pharmacology, Cumming School of Medicine, University of Calgary 70401, Calgary, Alberta, Canada; 5 Department of Biological Sciences, Faculty of Sciences, University of Calgary 98634, Calgary, Alberta, Canada; University of California San Diego School of Medicine, La Jolla, California, USA

**Keywords:** cathelicidin, *Toxoplasma gondii*, liver, brain

## Abstract

Toxoplasmosis, a disease caused by apicomplexan *Toxoplasma gondii (Tg)*, is associated with various neuropsychiatric and behavioral conditions. Toxoplasmosis can cause serious complications for those with weakened immune systems and during pregnancy. Cathelicidins, peptides with antimicrobial and immunomodulatory functions, are critical factors in host defense against microbes, but their role in parasitic infections is less well understood. This study demonstrates the protective function of endogenous cathelicidin against hepatic damage caused by *Tg* infection in an oral infection model. We challenged wild-type (*Camp^+/+^*) and cathelicidin-deficient (*Camp^-/-^*) mice with low-virulent Type II strain of *Tg* (ME-49) cysts. Our findings demonstrate that *Camp^-/-^* mice exhibited more severe clinical manifestations, higher mortality rates, and more pronounced hepatic damage compared to their *Camp^+/+^* counterparts. Histological liver examinations indicated significant necrotic hepatitis in *Camp^-/-^* mice, correlating with increased local concentrations of pro-inflammatory cytokines and proteomic upregulation of poly(ADP-ribose) polymerase 3 and guanylate-binding proteins. Increased cerebral inflammation and *Tg* cystogenesis were also observed in *Camp^-/-^* mice. Systemically, *Camp^-/-^* mice presented elevated levels of pro-inflammatory mediators, specifically interferon-gamma (Ifn-γ) and tumor necrosis factor-alpha (Tnf-α). In cultured macrophages, endogenous cathelicidin increased after *Tg* challenge, while *Camp^-/-^* bone marrow-derived macrophages released higher amounts of Tnf-α than their counterparts. We conclude that cathelicidin protects against liver injury and systemic deterioration induced by *Tg* infection by downregulating the synthesis of pro-inflammatory cytokines.

## INTRODUCTION

Toxoplasmosis, the disease caused by the protozoan apicomplexan *Toxoplasma gondii* (*T. gondii, Tg*), is highly prevalent worldwide (1, 2). *Tg* has a complex life cycle that enables it to infect all warm-blooded animals, including humans, which serve as intermediate hosts, while members of the *Felidae* family, such as domestic cats, function as definitive hosts (1, 2). Toxoplasmosis is typically acquired by ingesting tissue cysts found in infected meat or oocysts from food contaminated with cat feces (3). The rapid proliferation of tachyzoites marks the initial stages of infection and the onset of acute disease. These tachyzoites migrate from the initial infection site in the intestine to various tissues via the bloodstream and lymphatic systems, crossing the blood–brain barrier to reach the central nervous system (CNS) (4). In immunocompetent individuals, tachyzoites transform into bradyzoites, which replicate slowly and develop into cysts, thereby evading the immune response and perpetuating the infection in the host.

Toxoplasmosis can manifest differently among patients. Immunocompetent individuals are often asymptomatic, but *Tg* establishes a chronic, lifelong infection in the CNS. Other patients experience parasite reactivation, with severe acute disseminated toxoplasmosis and septic shock-like symptoms. Acute toxoplasmosis and eventual death can occur when the host’s immune defenses fail to control parasite activation and replication, such as in immunosuppressed patients, or after immunosuppressive treatments.

Critical innate immune effectors involved in host–parasite interactions and the immune response mechanisms that control *Tg* infection have often been overlooked. This is the case with cathelicidin, a short cationic antimicrobial peptide composed of an N-terminal domain that harbors a signal peptide, a conserved central cathelin domain, and variable C-terminal domains that exhibit antimicrobial activity (5). The only human cathelicidin is the 37-amino-acid-long C terminus (LL-37) (6), which is synthesized as a propeptide and cleaved extracellularly into the cathelin and the C-terminal LL-37 domains. Its murine counterpart is the cathelicidin-related antimicrobial peptide (CRAMP) encoded by the *Camp* gene (7). Secreted by neutrophils, macrophages, monocytes, myeloid bone marrow cells, and epithelial cells, cathelicidin has demonstrated microbicidal effects by disrupting microbial membranes and enhancing innate defenses through modulating cytokine secretion (8), chemotactic responses (9), pathogen recognition (10), and immune system activation (11). These functions contribute to processes in homeostasis, inflammation (12), wound healing (13), angiogenesis (14), autoimmune diseases (15), and cancer development (16). Thus, endogenous cathelicidin protects mice from colitis by regulating the recruitment and activation of monocytes (17). In addition, mice lacking cathelicidin exhibit more severe cutaneous infections with protozoan *Leishmania* (18), increased susceptibility to *Pseudomonas aeruginosa*-induced keratitis (19), and heightened vulnerability to *Helicobacter pylori* infection (20). This study aimed to determine whether cathelicidin provides host immune protection during toxoplasmosis, using an oral *Tg* infection model in cathelicidin-deficient (*Camp^-/-^*) mice compared to wild-type (*Camp^+/+^*) mice. The study reports that cathelicidin protects mice from severe systemic inflammation, particularly in the liver and brain, thereby preventing them from succumbing to *Tg* infection.

## MATERIALS AND METHODS

### Murine model of oral toxoplasmosis

Mice experiments were conducted following the Canadian Guidelines for Animal Welfare and were reviewed and approved by the University of Calgary Health Sciences Animal Care Committee (AC17-0078). We utilized male 6- to 8-week-old wild-type *Camp^+/+^* and cathelicidin-null *Camp^-/-^* C57BL/6 mice (B6.129X1-*Camp^tm1Rlg^*/J; The Jackson Laboratory). *Camp^-/-^* mice are homozygous for the targeted mutation, which disrupts exons 3 and 4 using a targeting vector containing a PGK-neo cassette. *Camp^-/-^* mice are viable, fertile, and regular in size and do not display any gross physical or behavioral abnormalities. Mice were housed in a pathogen-free environment in HEPA-filtered cages under a 12-h light cycle, with *ad libitum* access to sterilized feed and autoclaved water.

A Type II strain of *Tg* (ME-49) was used for the oral toxoplasmosis model. Tachyzoites of *Tg* were maintained *in vitro* by serial passage on monolayers of Vero cells incubated at 37°C in 5% CO_2_. Vero cells were grown in Dulbecco’s Modified Eagle Media supplemented with 10% fetal bovine serum (FBS, Gibco) and 1% penicillin and streptomycin (Gibco). *Camp*^+/+^ and *Camp*^-/-^ mice were orally gavaged with five cysts per mouse of *Tg* or phosphate-buffered saline (PBS) in sham mice. The relatively low *Tg* inoculum (five cysts) compared to other studies (>10 cysts), which usually provoke overproduction of Th-1 cytokines, intestinal necrosis, and death by 7 days post-infection (21–23), aimed to enable assessment of host immune responses without killing the host. Body weight and animal behavior were monitored daily, and mice were euthanized 14 days after the challenge. Starting around day 10 post-infection, *Camp^-/-^* mice exhibited significant weight loss and lethargy. Based on the veterinarian’s recommendation, a recovery gel (a water-based, cream-like nutritional supplement) was provided to the *Camp^-/-^* mice to encourage feeding and hydration. To maintain experimental consistency, the same recovery gel was subsequently provided to all groups (WT and PBS controls) for the remainder of the study. Humane endpoints were strictly followed, and mice showing >20% body weight loss or severe symptoms were euthanized according to institutional animal welfare protocols. Colon, ileum, liver, spleen, and brain were aseptically excised and sampled for histological analysis, gene transcription quantification, proteomics, and quantification of *Tg* burden.

### Macrophage cell models

To determine the impact of cathelicidin on the kinetics of human monocytes/macrophages infected with *Tg*, human monocyte-like (THP-1) cells differentiated into phagocytic cells were used. THP-1 cells were cultured in Roswell Park Memorial Institute 1640 (RPMI) supplemented with 10% FBS (Gibco), 1% penicillin and streptomycin (Gibco), and 0.1% 55 mM β−2-mercaptoethanol (Gibco) and differentiated into macrophage-like cells by adding phorbol myristate acetate (PMA; P8139, Sigma; 50 ng/mL) for 2 days. Non-attached cells were removed by aspiration, and adherent cells were washed with (1×) PBS (Gibco).

To determine the roles of endogenous cathelicidins in *Tg* infection, bone marrow-derived macrophages (BMDMs) from *Camp*^+/+^ and *Camp*^-/-^ mice were used. BMDMs were isolated using the Cold Spring Harbor protocol (24). Mice were euthanized, and bone marrow was collected from the femur and centrifuged (300 × *g*, 5 min). The resulting bone marrow pellet was suspended in BMDM media, composed of RPMI 1640 media supplemented with 10% FBS 1% penicillin and streptomycin, 0.1% 55 mM β-2-mercaptoethanol, and 10% mouse fibroblast (L-929)-conditioned media. The latter was used as a source of macrophage-colony stimulating factor (M-CSF) and included mouse fibroblasts (L-929; 5 × 10^5^) seeded in a T75 culture flask with 50 mL of THP-1 media (described previously) and incubated for 7 days at 37°C in a 5% CO_2_ atmosphere. The supernatant containing stimulating factors was collected and filtered twice, initially with a 45-μm filter (97066-206, VWR) and then with a 20-μm filter (97066-200, VWR). Bone marrow was seeded in 12-well plates with BMDM media for 6 days to promote differentiation. Every 2 days, unattached cells were removed by washing with (1×) PBS, and the BMDM media was replaced. After 6 days, the cells were fully differentiated into macrophages, as indicated by the attachment of the BMDM monolayer to the flask bottom.

Human PMA-differentiated THP-1 (dTHP-1) cells or murine BMDMs were plated (2 × 10^5^ cells per well) in 12-well plates (3513, Corning) and challenged with tachyzoites of *Tg* (Type II ME 49) at a multiplicity of infection (MOI) of up to 5:1 for 4 h. To prevent cell starvation during parasite infection, the media were supplemented with 2% FBS, 1% penicillin and streptomycin, and 0.1% 55 mM β-mercaptoethanol. dTHP-1 cells and murine BMDMs were stimulated apically with synthetic LL-37 peptides (H7298, Bachem; 1–2 μM) for 4 h during *Tg* infection. Cell survival and potential cytotoxicity were assessed by measuring lactate dehydrogenase (LDH) release from damaged cells. For this, an aliquot (50 μL) of supernatant from cultured cells was collected after parasite infection or exogenous LL-37 treatment, and LDH concentrations were determined using a Pierce LDH Cytotoxicity Assay (88953, Thermo Fisher Scientific). Supernatants (1 mL) from uninfected and *Tg*-infected human (dTHP-1) or BMDMs were collected and stored at −80°C. Concentrations of TNF-α, IFN-γ, and IL-1β in human or murine cell culture supernatants were measured using a sandwich enzyme-linked immunosorbent assay (ELISA) (DuoSet; R&D Systems). Similarly, concentrations of LL-37 in dTHP-1 cell culture supernatants were measured with an ELISA (HK321-02; Hycult Biotech).

### Histology in murine organs

Samples of colon, ileum, liver, spleen, and brain were immediately fixed in 10% neutral buffered formalin (89370-094, VWR) for 1 day and dehydrated in 70% ethanol, using a semi-enclosed benchtop tissue processor (TP1020, Leica) before being embedded in paraffin. Sections were cut (5 μM), mounted on slides, and stained with hematoxylin (6765008, Thermo Fisher Scientific) and eosin (6766008, Thermo Fisher Scientific). Conventional histological evaluation was performed on all sections with the assistance of a pathologist, Dr. Cameron Knight (Faculty of Veterinary Medicine, University of Calgary). The total necrotic areas of the liver were quantified using ImageJ for morphometric analysis of hematoxylin and eosin-stained sections (25). Digital photomicrographs of each necrotic focus in the liver were captured at 10× magnification. Images were converted to 8-bit grayscale, and the “threshold” function was applied to detect and measure relevant areas, excluding empty spaces. Thus, the location of each lesion was calculated to determine the average size of the necrotic center, and these values were summed with those from other regions to assess the total number of focal lesions in each sample. The percentage of necrotic tissue was calculated by dividing the area affected by the total area of the section, measured with a ruler as a reference.

### Transcriptional gene expression of innate factors

Frozen tissues were homogenized for the quantification of gene transcription of immune effectors (26). Relative messenger RNA (mRNA) expression of mouse cathelicidin (cathelicidin-related antimicrobial peptide; *Camp*) and cytokines (*Tnf-α*, *Ifn-γ,* and *Il-1β*) from murine tissues, as well as human cathelicidin/LL-37 (cathelicidin antimicrobial peptide; *CAMP*) and cytokines (*TNF-α, IFN-γ*) from dTHP-1 macrophages, were quantified by reverse transcription real-time polymerase chain reaction (RT-qPCR). Total RNA was isolated using TRIzol reagent (Invitrogen). Complementary DNA (cDNA) was prepared from 1 μg of total RNA using Moloney murine leukemia virus reverse transcriptase (iScript Reverse Transcription Supermix for RT-qPCR, Bio-Rad). The quality and quantity of the resulting RNA and cDNA were assessed using a spectrophotometer (NanoVue, GE Healthcare). The absence of contaminating genomic DNA in RNA preparations was verified using a minus-reverse transcriptase control (i.e., a sample containing all RT-PCR reagents except reverse transcriptase). RT-qPCR was performed using a CFX-96 real-time PCR system (Bio-Rad). Each reaction mixture consisted of 100 ng of cDNA, SYBR Green Real-Time PCR Master Mix (Thermo Fisher Scientific), and 0.5 μM of each specific primer, for a final volume of 10 μL. Primer pairs (330001, RT^2^ qPCR Primer Assay, Qiagen) were used for murine *Camp*, *Il1-β, Tnf-α,* and *Ifn-γ*, as well as human *CAMP*, *TNF-α,* and *IFN-γ*, and were verified for specificity and efficiency (Table 1). The RT-qPCR efficiency for each gene was calculated from the slope of a linear regression model using the equation: efficiency = 10^(−1/slope)^ − 1, as indicated in the MIQE guidelines (27). *R*² values were calculated using 10-fold serial dilutions of cDNA. Reaction mixtures were incubated at 95°C for 5 min, followed by denaturation for 5 s at 95°C and combined annealing/extension for 10 s at 60°C for 40 cycles. Two housekeeping genes, *GAPDH* and *β-actin*, were initially tested; neither exhibited alterations, and *GAPDH* was selected. Negative controls for cDNA synthesis and PCR procedures were consistently included. The target mRNA values were corrected relative to the housekeeping gene *GAPDH*. Data were analyzed using the 2^−ΔΔCT^ method. Results reported the mean fold change in target transcription levels between *Tg*-infected groups and the sham/PBS group.

**TABLE 1:** Details of primers for mRNA relative quantification of immune effectors by real-time RT-PCR

Symbol	Catalog	RefSeq^*a*^	Description	*R* ^2^
*Gapdh*	PPM02946E	NM_008084	Mouse glyceraldehyde-3-phosphate dehydrogenase	0.9517
*Ifn-γ*	PPM03121A	NM_008337	Mouse interferon, gamma	0.9517
*Tnf-α*	PPM03113G	NM_013693	Mouse tumor necrosis factor	0.9517
*Camp*	PPM25023A	NM_009921	Mouse cathelicidin antimicrobial peptide	0.9517
*Il1-b*	PPM03109F	NM_008361	Mouse interleukin 1 beta	0.9517
*GAPDH*	PPH00150F	NM_002046.5	Human glyceraldehyde-3-phosphate dehydrogenase	0.99982
*IFN-γ*	PPH00380C	NM_000619.2	Human interferon, gamma	0.98308
*TNF-α*	PPH00341F	NM_000594.3	Human tumor necrosis factor	0.99899
*CAMP*	PPH09430A	NM_004345.3	Human cathelicidin antimicrobial peptide	0.9999

^*a*^ RefSeq accession number: the sequence used to design the RT2 qPCR Primer Assay.

### Detection of secreted cytokines from murine blood plasma

Plasma cytokines in mice were assessed by collecting a cardiac blood sample. Heparinized blood samples (0.1–0.2 μL of 1×, one heparin unit) were centrifuged (1,300 × *g* 10 min at 4°C), transferred into a clean tube, and stored at −80°C. Blood plasma was diluted 1:2 with sterile dPBS. Cytokine concentrations (GM-CSF, Ifn-γ, Il-1β, Il-2, Il-4, Il-6, Il-10, Il-12p70, Mcp-1, Tnf-α) were quantified by a Luminex Multiplex Mouse Cytokine Array Proinflammatory Focused 10-plex (MDF 10; Eve Technologies Discovery Assay).

### Detection of *Tg* in tissues

*Tg* was quantified in immediately snap-frozen murine organs (colon, ileum, liver, spleen, and brain) and macrophage cell lysates. *Tg* genomic DNA was extracted from tissues (20–30 mg) and cell supernatants (E.Z.N.A. Tissue DNA Kit, D3396-02, Omega Bio-tek). The burden of *Tg* was quantified by assessing the constitutively expressed multicopy B1 gene of *Tg* (GenBank AF179871) (Table 2) (28) by RT qPCR using a CFX-96 real-time PCR system (BioRad). Each reaction mixture contained 100 ng of gDNA, SYBR Green Real-Time PCR Master Mixes (Thermo Fisher Scientific), and 0.5 μM of each specific primer in a final volume of 10 μL. A positive standard curve with increasing concentrations of *Tg* DNA, indicative of amounts of *Tg* tachyzoites (ME 49), was derived by counting tachyzoites with phase-contrast microscopy in a counting chamber and extracting DNA from the tachyzoite suspension.

**TABLE 2:** Details of primers for mRNA relative quantification of *Tg* by real-time RT-PCR

Symbol	Sequences	RefSeq^*a*^	Description	Reference
B1	F: 5'TCCCCTCTGCTGGCGAAAAGT-3′	MH814767	*Toxoplasma gondii* 2TGILZSP/PA B1 gene, intron	(29)
	R: 5'AGCGTTCGTGGTCAACTATCGATTG-3′			

^*a*^ RefSeq accession number: the sequence used to design the RT^2^ qPCR Primer Assay.

### Detection of *Tg* cysts

Cysts of *Tg* in the liver and brain from uninfected and *Tg*-infected *Camp*^+/+^ or *Camp*^-/-^ mice were detected by immunohistochemistry. Tissue sections were deparaffinized and hydrated using an auto stainer (Agilent Technologies). Epitopes were retrieved by treating the sample with proteinase K (Proteinase K, Agilent Technologies) for 30 min, followed by a 30-min incubation with the primary goat anti-*Tg* antibody (1:5,000) (VMRD). The binding of the primary antibody was revealed using rabbit anti-goat immunoglobulins (Vector Labs) and an avidin-biotin immunoperoxidase complex reagent, with 3,3′-diaminobenzidine tetrahydrochloride (DAB) as the chromogen (Dako Liquid DAB+ Substrate Chromogen System, Agilent Technologies Canada Inc.). Sections were counterstained with Mayer’s hematoxylin. Ten random fields were selected for each slide, and a sample was considered positive when DAB-stained bradyzoite pseudocysts were brown. Mice were considered cyst-positive by IHC when at least one of the evaluated organs was positive. The total number of cysts was counted in each mouse group.

### Proteomic analysis of the liver

Livers from mice were subjected to quantitative shotgun proteomic analysis using liquid chromatography and tandem mass spectrometry (LC-MS/MS). Liver samples were lysed in a buffer composed of 1% SDS, 200 mM HEPES (pH 8.0), 100 mM ammonium bicarbonate, 10 mM EDTA, and protease inhibitor complete tablets (Roche, Mannheim, Germany). Disulfide bonds of 100 μg of total protein were reduced with 10 mM Tris (2-carboxyethyl) phosphine hydrochloride (Thermo Fisher Scientific) at 55°C for 1 h. The proteins were alkylated by incubation with 15 mM iodoacetamide (VWR) for 25 min in the dark at room temperature. Proteins were precipitated from the solution by adding 600 μL of ice-cold acetone and incubated at 20°C overnight. Samples were centrifuged at 8,000 × *g* for 10 min before resuspension in 100 μL of 50 mM triethyl ammonium bicarbonate. Proteins were trypsinized (Thermo Fisher Scientific) overnight at a 1:10 enzyme-to-substrate ratio. For tandem mass tag (TMT) 6-plex labeling (TMTsixplex isobaric label reagent set, Thermo Fisher Scientific), 0.8 mg of TMT reagent was resuspended in 41 μL of acetonitrile, and samples were spun down quickly at 2,000 rpm (380 g) for 10 s and incubated at room temperature for 1 h. A total of four biological replicates per group (*n* = 4 mice per group) were labeled with TMT reagents and one TMT tag (131) contained the pooled samples (i.e., equal amounts of peptides [20 µg] of each sample per group) and served as internal standards for normalizing the data across the groups. For labeling, peptides were incubated with TMT reagents (room temperature, 1 h), and the reaction was quenched by adding 8 μL of 5% hydroxylamine and incubated for 15 min at 25°C. Peptides with different labels were combined before 100% formic acid was added to each sample to reach a volumetric concentration of 1% formic acid. Samples were spun at 5,000 rpm (2,350 × *g*) for 10 min and desalted using Sep-Pak C18 columns (Waters, 130 mg WAT023501). Sep-Pak columns were conditioned with 1 × 3 mL 90% methanol/0.1% TFA, 1× 2 mL 0.1% formic acid. Each sample was loaded onto a column and washed with 1 × 3 mL 0.1% TFA/5% methanol. Peptides were eluted from the column with 1 × 1 mL 50% ACN/0.1% formic acid and lyophilized. Peptides were resuspended in 1% formic acid, and a BCA assay (Thermo Fisher Scientific) was used to determine the peptide concentration in each sample. Samples were dried down and stored at −80°C. LC-MS/MS experiments were performed on an Orbitrap Fusion Lumos Tribrid mass spectrometer (Thermo Fisher Scientific) operated with Xcalibur (v4.0.21.10) and coupled to a Thermo Scientific Easy-nLC (nanoflow Liquid Chromatography) 1200 system. Tryptic peptides (2 μg) were loaded onto a C18 trap (75 μm × 2 cm; Acclaim PepMap 100, P/N 164946; Thermo Fisher Scientific) at a flow rate of 2 μL/min of solvent A (0.1% formic acid and 3% acetonitrile in LC mass spectrometry grade water). Peptides were eluted using a 120-min gradient from 5% to 40% (5%–28% in 105 min followed by an increase to 40% B in 15 min) of solvent B (0.1% formic acid in 80% LC-mass spectrometry grade acetonitrile) at a flow rate of 0.3 μL/min and separated on a C18 analytical column (75 µm × 50 cm; PepMap RSLC C18; P/N ES803; Thermo Fisher Scientific). Peptides were electrosprayed using 2.3 kV voltage into the ion transfer tube (300°C) of the Orbitrap Lumos operating in positive mode. The Orbitrap first performed a full mass spectrometry scan at a resolution of 120,000 FWHM to detect precursor ions with a mass-to-charge ratio (m/z) between 375 and 1,575 and a charge state range of +2 to +4. The Orbitrap AGC (Auto Gain Control) and the maximum injection time were set to 4 × 10^5^ and 50 ms, respectively. The Orbitrap was operated in top-speed mode with a 3-s cycle time for precursor selection. The most intense precursor ions presenting a peptidic isotopic profile and having an intensity threshold of at least 2 × 10^4^ were isolated using the quadrupole (isolation window of m/z 0.7) and fragmented with HCD (38% collision energy) in the ion routing Multipole. The fragment ions (MS2) were analyzed in the Orbitrap at a resolution of 15,000. The AGC, maximum injection time, and the first mass were set at 1 × 10^5^, 105 ms, and 100, respectively. Dynamic exclusion was enabled for 45 s to prevent the acquisition of duplicate precursor ions with similar m/z values (±10 ppm). For bioinformatics analysis, spectral data were matched to peptide sequences against *Mus Musculus* UniProt (UniProt, 2023) protein database using the Andromeda algorithms (30) implemented in the MaxQuant (31) software package v.1.6.0.1, at a peptide-spectrum match false discovery rate (FDR) of <0.01. Search parameters included a mass tolerance of 20 ppm for the parent ion, 0.5 Da for the fragment ion, carbamidomethylation of cysteine residues (+57.021464 Da), variable N-terminal modification by acetylation (+42.010565 Da), and variable methionine oxidation (+15.994915 Da). TMT 6-plex labels 126–131 were designated for relative quantification. The cleavage site specificity was set to trypsin/P (searching for free N-termini and for only lysine), with up to two missed cleavages allowed. The normalized results for each group were averaged, followed by the ratio for each group comparison. The ratios were log_(2)_-transformed, and the significant outlier cutoff values were determined after log_(2)_ transformation by boxplot-and-whiskers analysis using the BoxPlotR tool.

### Statistical analyses

Graphs represent normally/Gaussian distributed (parametric) results presented as means and standard errors of the mean derived from independent experiments. Normality was assessed using the Shapiro-Wilk test (Royston [1]). All comparisons were performed using paired two-tailed Student’s *t*-tests between treated groups and the control group or differences among groups by analysis of variance (ANOVA), with Bonferroni post-tests. Differences were considered statistically significant for *P* < 0.05. All statistical analyses were performed with GraphPad Prism software (GraphPad 8.0). Proteomic bioinformatic analysis, GO annotation, proteins’ basic functions, domain functional descriptions, and annotation of biological processes were studied using the UniProt-GOA database (http://www.ebi.ac.uk/GOA/), InterPro domain database (http://www.ebi.ac.uk/interpro/), and the Kyoto Encyclopedia of Genes and Genomes (KEGG) database (http://www.genome.jp/kaas-bin/kaas_main; http://www.kegg.jp/kegg/mapper.html). A two-tailed Fisher’s exact test was employed to test the enrichment of the differentially expressed proteins (DEPs) against all identified proteins. All the categories obtained after enrichment were collected and filtered for those at least enriched in one of the clusters with a *P*-value < 0.05. The database accession numbers or sequences of all DEPs were searched against the STRING database (v11) for protein–protein interaction analysis. *P*-values of <0.05 were considered statistically significant for all analyses (MaxQuant software [v.1.6.0.1]) using a peptide FDR of 0.01. Data were implemented and analyzed in the Python environment. Hierarchical clustering was performed, and the heatmap was plotted using Seaborn (v0.11.1). Other packages, including matplotlib, scikit-learn, pandas, and NumPy, were also used to process and plot the results.

## RESULTS

### *Camp^-/-^* mice succumbed to oral *Tg* infection

**Fig 1:**
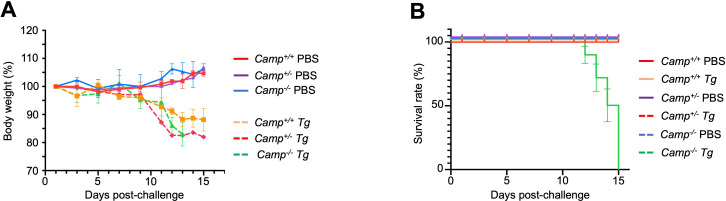
*Camp*^-/-^ mice reached a critical human endpoint during oral infection with *Tg* cysts. (**A and B**) *Camp*^+/+^ and *Camp*^-/-^ mice were orally gavaged with five cysts of *Tg* (ME-49 strain, *Tg*) or sham PBS. (**A**) Mice were weighed daily and euthanized 14 days after infection. (**B**) Survival was daily monitored, and *Camp*^-/-^ *Tg*-infected mice succumbed by 12 days post-infection, whereas *Camp*^+/+^-infected mice and PBS mice continued throughout the study. Data are shown as means ± SEM (*n* = 4–6 mice per group).

The role of endogenous cathelicidin in toxoplasmosis was examined in *Camp^+/+^* and *Camp^-/-^* mice using a natural infection model based on an oral infection with oocysts from a low virulent Type II isolate, *Tg* (ME 49), which exhibited remarkable morbidity (21–23). We showed homozygous *Camp*^-/-^ mice infected by *Tg* displayed more severe clinical manifestations of illness by day 9 post-challenge, including lethargy and hirsute fur. They lost more weight (~20%) compared to their *Camp*^+/+^ (~10%) littermates (*P* > 0.05, Fig. 1A). Furthermore, *Camp*^-/-^ *Tg*-infected mice reached the human critical endpoint earlier, experiencing weight loss (>20%) before 14 days post-challenge, while *Camp*^+/+^-infected mice survived throughout the study (Fig. 1B). Both *Camp*^+/+^ and *Camp*^-/-^ mice inoculated with PBS (control) survived without clinical signs and showed consistent daily weight gain (*P* > 0.05, Fig. 1A and B).

### Cathelicidin protected mice from hepatitis after oral challenge with *Tg*

**Fig 2:**
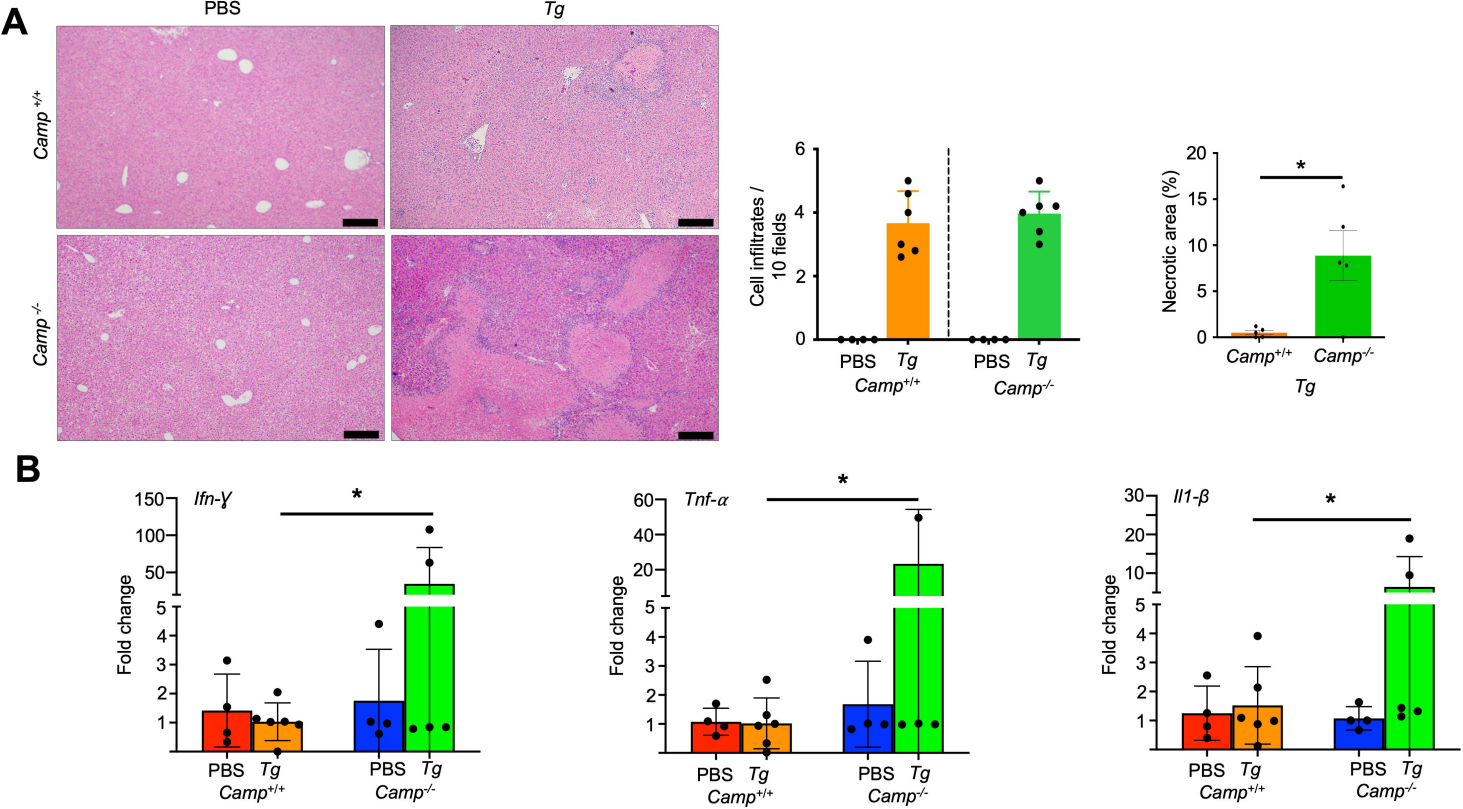
*Camp*^-/-^ mice orally infected with *Tg* cysts increased the production of pro-inflammatory cytokines in the inflamed liver. (**A**) Microphotography and histological scoring of lesions in H&E-stained sections of livers of *Camp*^+/+^ and *Camp*^-/-^ infected with *Tg* (ME-49 strain, *Tg*) and sham PBS mice (*n* = 4–6 mice per group). Cell infiltrations in quantified 10 random fields were similarly observed in both *Camp*^+/+^ and *Camp*^-/-^ *Tg*-infected mice, but the *Camp*^-/-^-infected liver showed more necrotic areas. Bar scale: 100 µm. (**B**) Transcriptomic expression of *Ifn-γ*, *Tnf-α,* and *Il1-β* genes in the livers of *Camp*^+/+^ and *Camp*^-/-^ *Tg*-infected and sham mice. It shows a trend toward higher cytokine expression in *Camp*⁻/⁻ Tg-infected livers, although the differences did not reach statistical significance (*n* = 4–6 mice per group). Data are presented as mean ± SEM. *A *P* < 0.05 (two-tailed Student’s *t* test or one-way ANOVA with Tukey’s post hoc test for multiple comparisons) was considered statistically significant.

To account for sudden illness and death in *Camp*^-/-^-infected mice, we examined the liver, a key affected organ in toxoplasmosis (32). *Camp*^+/+^ and *Camp*^-/-^ *Tg*-infected mice showed some degree of cell infiltration in their livers, consisting of neutrophils, macrophages, and lymphocytes in the parenchyma after 14 days post-challenge (Fig. 2A). Livers from *Camp*^-/-^ *Tg*-infected mice displayed more pronounced diffuse Kupffer cell hyperplasia and severe multifocal necrotizing nonsuppurative hepatitis, characterized by central necrotic foci surrounded by macrophages, lymphocytes, and plasma cells (Fig. 2A). These necrotic areas in *Tg*-infected *Camp*^-/-^ livers were more extensive compared to those of their *Camp*^+/+^ counterparts (*P* < 0.05) (Fig. 2A). Inflammatory cytokines *Ifn-γ*, *Tnf-α*, and *Il1-β* showed a trend toward higher levels in *Camp*^⁻/⁻^ mice compared with both *Camp*^+^/^+^ and control groups (Fig. 2B).

**Fig 3:**
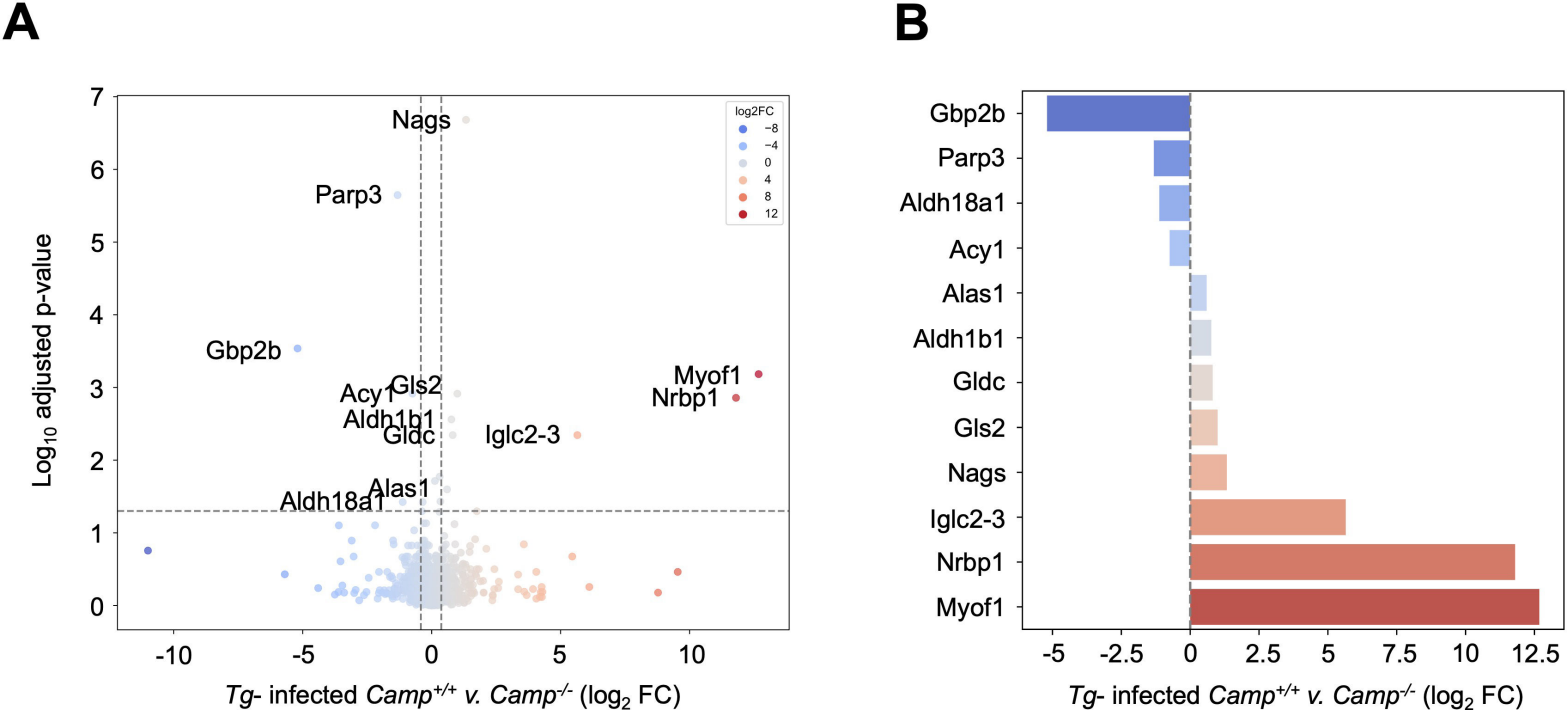
Hepatic proteomic profile in *Camp^+/+^* and *Camp^-/-^* mice challenged with *Tg. Camp*^+/+^ and *Camp*^-/-^ mice were orally gavaged with five cysts of *Tg* (ME-49, *Tg*) or sham PBS. Livers at 14 days post-*Tg* infections were analyzed by shotgun proteomics, and the differential enrichment protein/peptide analysis is shown as (**A**) a volcano plot and (**B**) fold change (FC) expression (*n* = 4 mice per group).

Since *Camp*^-/-^-infected mice exhibited increased hepatitis and elevated cytokine levels, we determined the hepatic proteomic profile of *Camp*^+/+^ and *Camp*^-/-^ mice at 14 days post-challenge with *Tg. Camp*^-/-^*Tg*-infected livers showed a significant upregulation of the p65 family guanylate-binding protein 1 (Gbp2b) (5.5-fold), poly (ADP-ribose) polymerase (PARP) 3 (3.5-fold), delta-1-pyrroline-5-carboxylate synthase (Aldh18a1), and aminoacylase-1 (Acy1) compared to their *Camp*^+/+^ counterparts (Fig. 3A and B; Tables S1 and S2). These data suggest that the absence of cathelicidin in *Camp*^-/-^ mice leads to an exaggerated hepatic response to *Tg* infection, thereby exacerbating disease severity and predisposing to systemic disturbances.

### No different inflammatory responses in systemic organs in mice lacking cathelicidin after oral challenge with *Tg*

**Fig 4:**
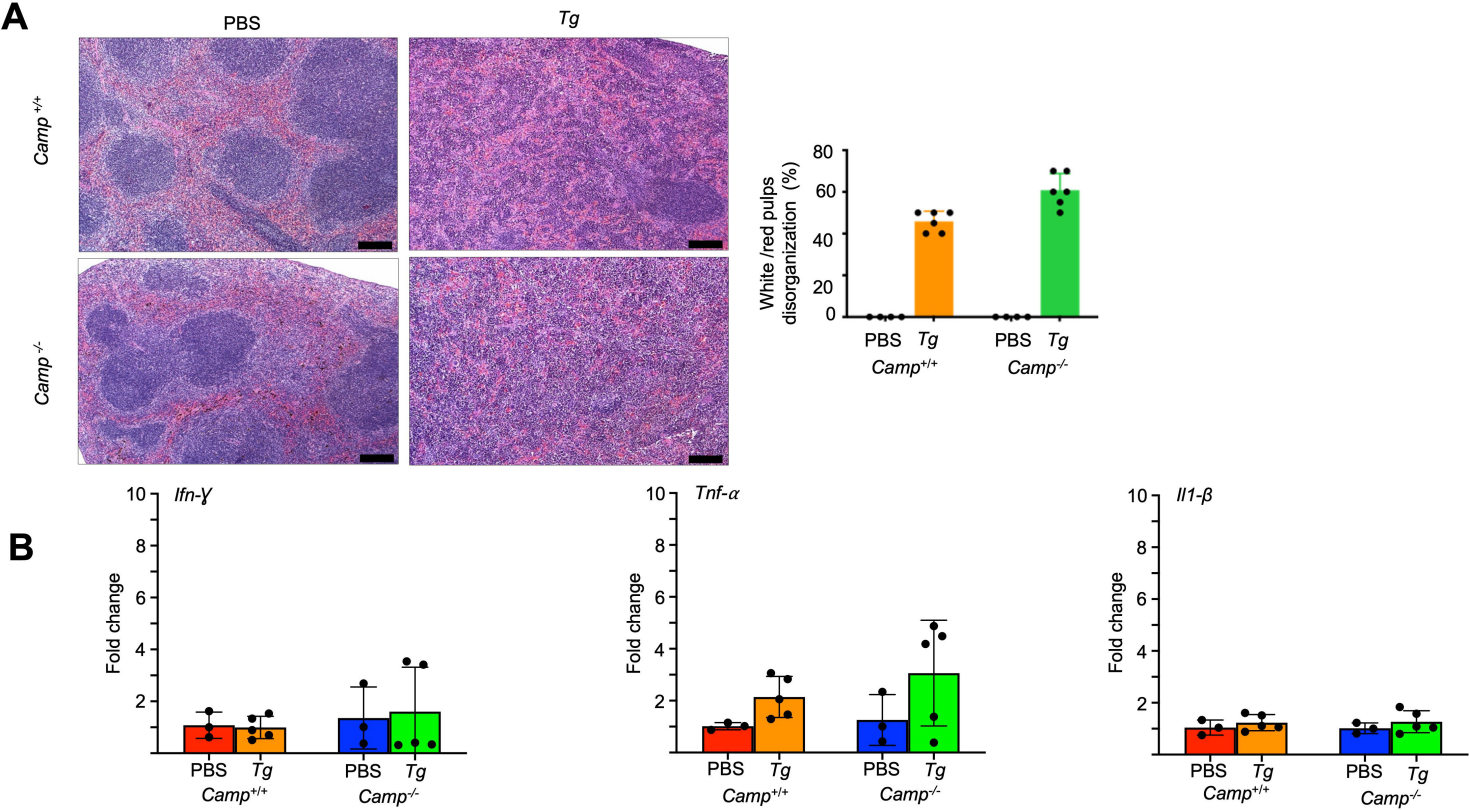
*Camp*^+/+^ and *Camp*^-/-^mice orally infected with *Tg* cysts show similar levels of splenitis. (**A**) Microphotography and histological scoring of lesions in H&E-stained sections of spleens of *Camp*^+/+^ and *Camp*^-/-^-infected *Tg* (ME-49 strain, *Tg*) and sham PBS mice (*n* = 4–5 mice per group). A well-defined structure of white and red pulps was observed in PBS control, whereas there was a similar spleen disruption in *Camp*^+/+^ and *Camp*^-/-^-infected spleens in quantified 10 random fields. Bar scale: 100 µm. (**B**) Transcriptomic expression of *Ifn-γ*, *Tnf-α,* and *Il1-β* genes in the spleens of *Camp*^+/+^ and *Camp*^-/-^ *Tg*-infected and sham mice (*n* = 4–6 mice per group). The differences were not statistically significant. Data are shown as means ± SEM.

As *Tg* spreads from the gut to become systemic via migration across the intestinal epithelium, we determined whether a *Tg* challenge in mice deficient in cathelicidin affects other systemic organs or promotes ileitis or colitis. *Camp*^+/+^ and *Camp*^-/-^ mice infected with *Tg* displayed comparable histological structural disruption in the spleen, exhibiting a marked loss of distinction between red and white pulps (Fig. 4A). Gene expression of *Ifn-γ*, *Tnf*-α, and *Il1-β* was also similar between *Camp*^⁻/⁻^ *Tg-*infected mice relative to wild-type counterparts (Fig. 4B).

**Fig 5:**
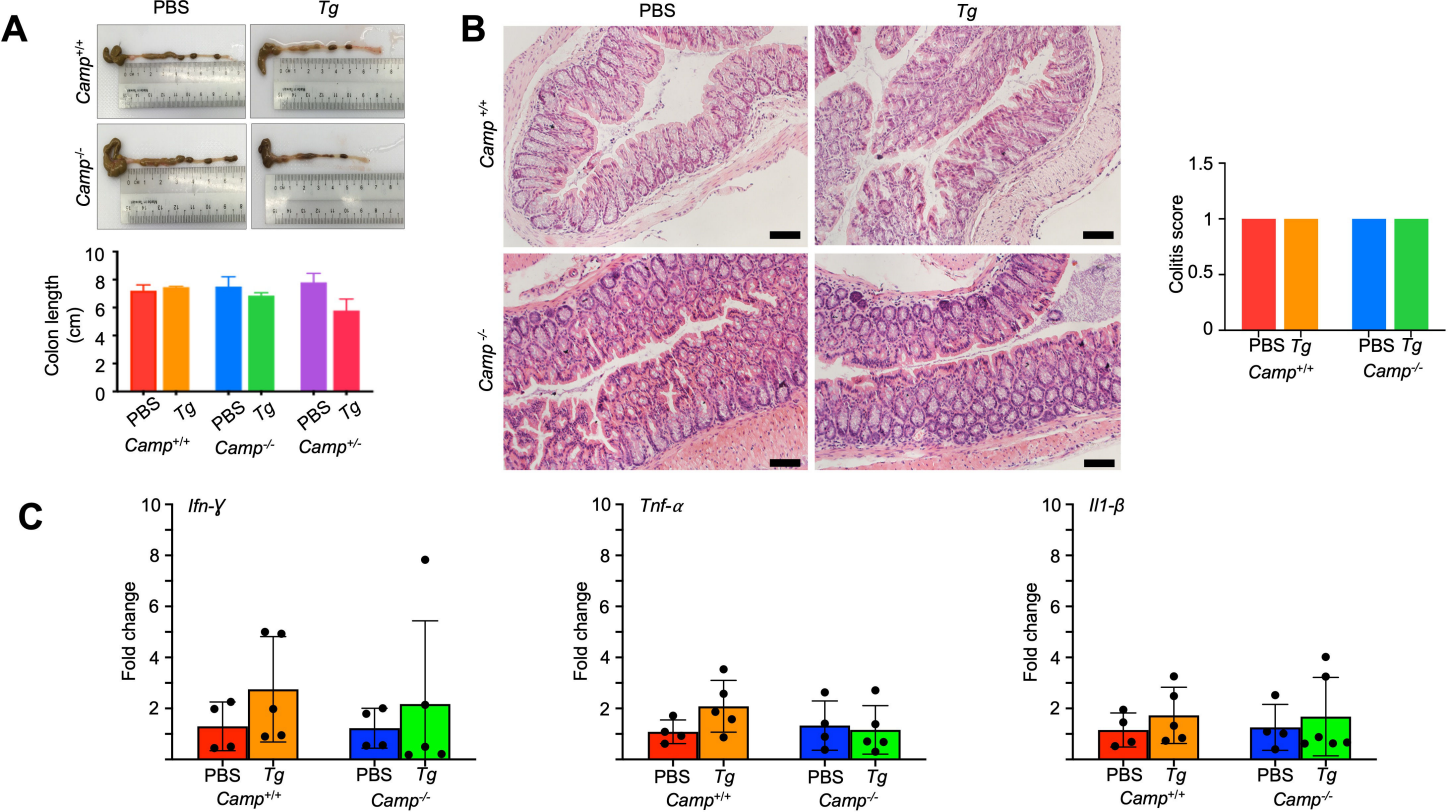
No signs of colitis in *Camp*^+/+^ and *Camp*^-/-^ mice orally infected with *Tg* cysts. (**A**) Length of colon with cecum in *Camp*^+/+^ and *Camp*^-/-^ mice orally infected with *Tg* (ME-49 strain, *Tg*) or sham PBS for 14 days (*n* = 5-6 mice per group) was imaged and measured with no significant differences. Data are shown as means ± SEM. (**B**) Microphotographs of the H&E-stained sections of colons of these mice revealed no signs of colitis (*n* = 4–5 per group). Bar scale: 50 µm. (**C**) Transcriptomic expression of *Ifn-γ*, *Tnf-α,* and *Il1-β* genes in the colons of *Camp*^+/+^ and *Camp*^-/-^ *Tg*-infected and sham mice (*n* = 4–5 mice per group). The differences were not statistically significant. Data are shown as means ± SEM.

**Fig 6:**
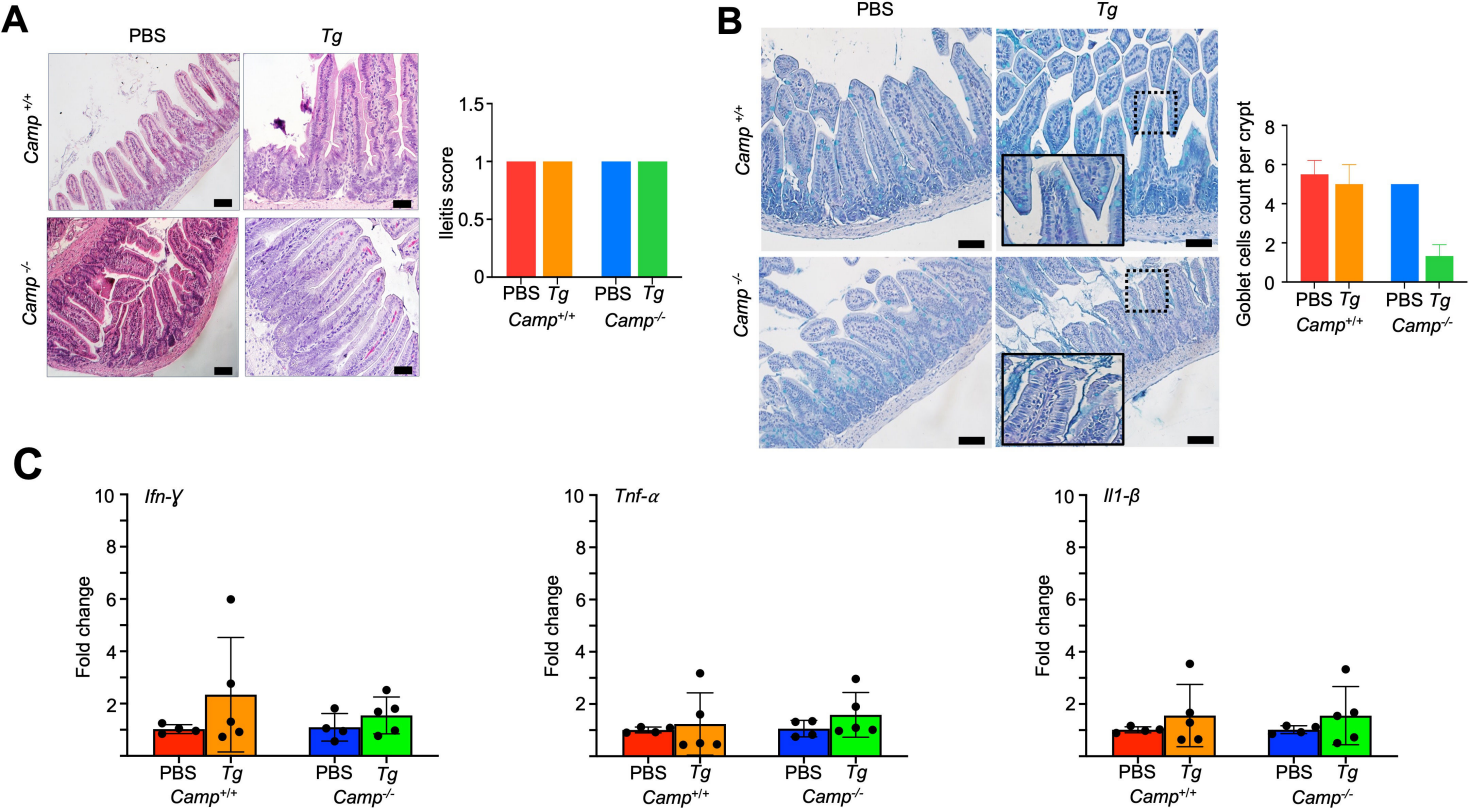
No signs of ileitis in *Camp*^+/+^ or *Camp*^-/-^ mice orally infected with *Tg* cysts. (**A**) Microphotographs of H&E-stained sections of the ileums of *Camp*^+/+^ and *Camp*^-/-^ mice orally infected with *Tg* (ME-49 strain, *Tg*) or sham PBS for 14 days (*n* = 5–6 mice per group) revealed no signs of ileitis. Bar scale: 50 µm. (**B**) Microphotographs of Alcian blue-stained ileum sections show no differential mucin barrier, although *Camp*^-/-^-infected ileums showed fewer goblet cells compared to *Camp*^+/+^ counterparts (*zoom in*) (*n* = 4–5 mice per group). Bar scale: 50 µm. (**C**) Transcriptomic expression of *Ifn-γ*, *Tnf-α,* and *Il1-β* genes in the ileums of *Camp*^+/+^ and *Camp*^-/-^ *Tg*-infected and sham mice (*n* = 4–5 mice per group). Data are shown as means ± SEM.

No shortening of colon length was observed in *Camp*^+/+^ or *Camp*^-/-^ *Tg*-infected mice compared to PBS control mice after 14 days post-challenge (*P* > 0.05, Fig. 5A). Likewise, colons from both *Camp^+/+^* and *Camp^-/-^ Tg*-infected mice, compared to PBS-treated control mice, showed no microscopic lesions, epithelial disruption, or immune cell infiltration (*P* > 0.05, Fig. 5B), nor any differences in gene transcription levels of pro-inflammatory cytokines *Ifn-γ*, *Tnf-α*, and *Il1-β* (*P* > 0.05, Fig. 5C). The ileum from *Camp*^+/+^ and *Camp*^-/-^ mice exposed to *Tg* showed no epithelial disruption or inflammatory cell infiltration (*P* > 0.05, Fig. 6A). Only a slight, not significant, loss of goblet cells was noted in the ileum of *Camp*^-/-^*Tg-*infected mice compared to those of *Camp*^+/+^-infected mice, as determined by Alcian blue staining (Fig. 6B). The expression of pro-inflammatory cytokine genes (*Ifn-γ*, *Tnf*-α, and *Il1-β*) in the ileum was similar in *Camp*^+/+^ and *Camp*^-/-^ mice infected with *Tg* and did not differ from uninfected mice (*P* > 0.05, Fig. 6C).

### Cathelicidin prevented the grade of *Tg* cyst formation and histological lesions in the brain

**Fig 7:**
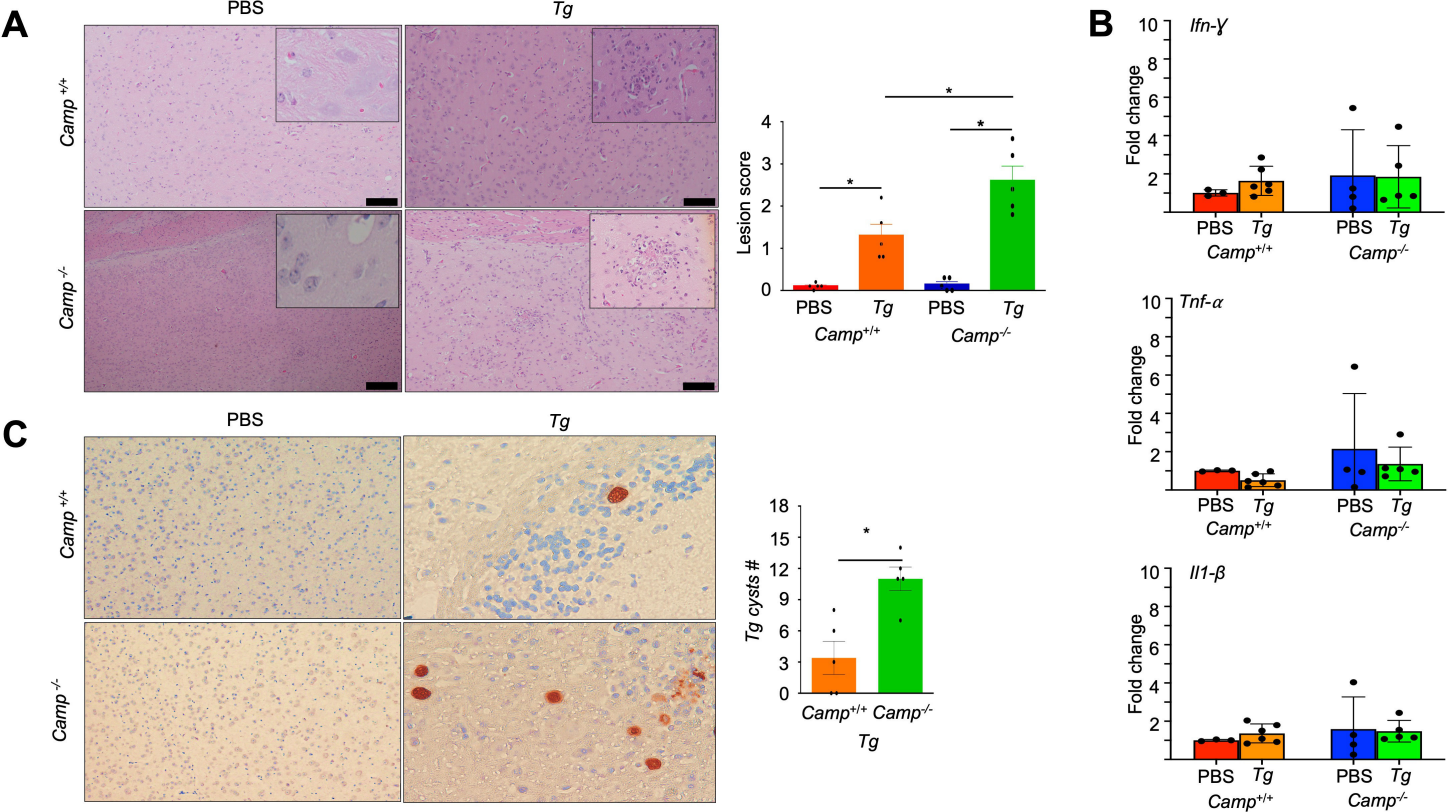
Similar encephalitis in *Camp*^+/+^ and *Camp*^-/-^ mice orally infected with *Tg* cysts. (**A**) H&E-stained brains of *Camp*^+/+^ and *Camp*^-/-^-infected mice with *Tg* (ME-49 strain, *Tg*) or sham PBS for 14 days (*n* = 4–5 mice per group), showing a similar number of cell infiltrates in 10 random fields, with no significant differences between *Camp*^+/+^ and *Camp*^-/-^ *Tg-*infected mice. Bar 100 µm. Data are shown as means ± SEM. *P < 0.05 (two-tailed Student’s t-test or one-way ANOVA *post hoc* Tukey’s test for multiple comparisons) was considered significant. (**B**) Transcriptomic expression of *Ifn-γ*, *Tnf-α,* and *Il1-β* genes in the brains of *Camp*^+/+^ and *Camp*^-/-^ *Tg*-infected and sham mice (*n* = 4–5 mice per group). Data are shown as means ± SEM. (**C**) Immunohistochemistry detection of B1 *Tg* (*n* = 5 mice per group). There was a higher number of *Tg* tachyzoites in the brains of *Camp*^-/-^ *Tg*-infected mice than *Camp*^+/+^-infected counterparts. Scale bar 50 μM. Data are shown as means ± SEM. *P < 0.05 (two-tailed Student’s t-test or one-way ANOVA *post hoc* Tukey’s test for multiple comparisons) was considered significant.

To persist lifelong within a host, *Tg* eventually invades the brain and establishes a quiescent, chronic infection (33). We showed that the brains of *Camp*^+/+^-infected mice had no lesions or inflammatory cell infiltration 14 days post-infection (Fig. 7A). In stark contrast, *Camp*^-/-^-infected mice exhibited nonsuppurative encephalitis, characterized by infiltration of lymphocytes, macrophages, and reactive astrocytes alongside substantial necrotic areas (Fig. 7A). Perivascular cuffing and vasculitis were also noted, with isolated tissue cysts within neurons without an associated inflammatory reaction (Fig. 7A). The transcriptional mRNA expression levels of *Ifn-γ*, *Tnf-α*, and *Il1-α* in the brains of *Camp*⁻/⁻ and *Camp*^+^/^+^ Tg-infected mice were comparable and did not differ significantly (*P* > 0.05, Fig. 7B). Despite similar cytokine profiles, *Tg*-positive cysts were detected in the brains of all infected *Camp*⁻/⁻ mice (5/5), whereas up to 40% of infected *Camp*^+^/^+^ mice showed no detectable cysts (3/5), resulting in a significantly higher cyst burden in the absence of cathelicidin (*P* < 0.05, Fig. 7C). These findings suggest certain immunological permissiveness in *Camp^-/-^ Tg*-infected mice that could allow rapid replication of tachyzoites and a higher parasite burden in the CNS.

### Cathelicidin prevents overwhelming inflammation but not *Tg*’s burden

**Fig 8:**
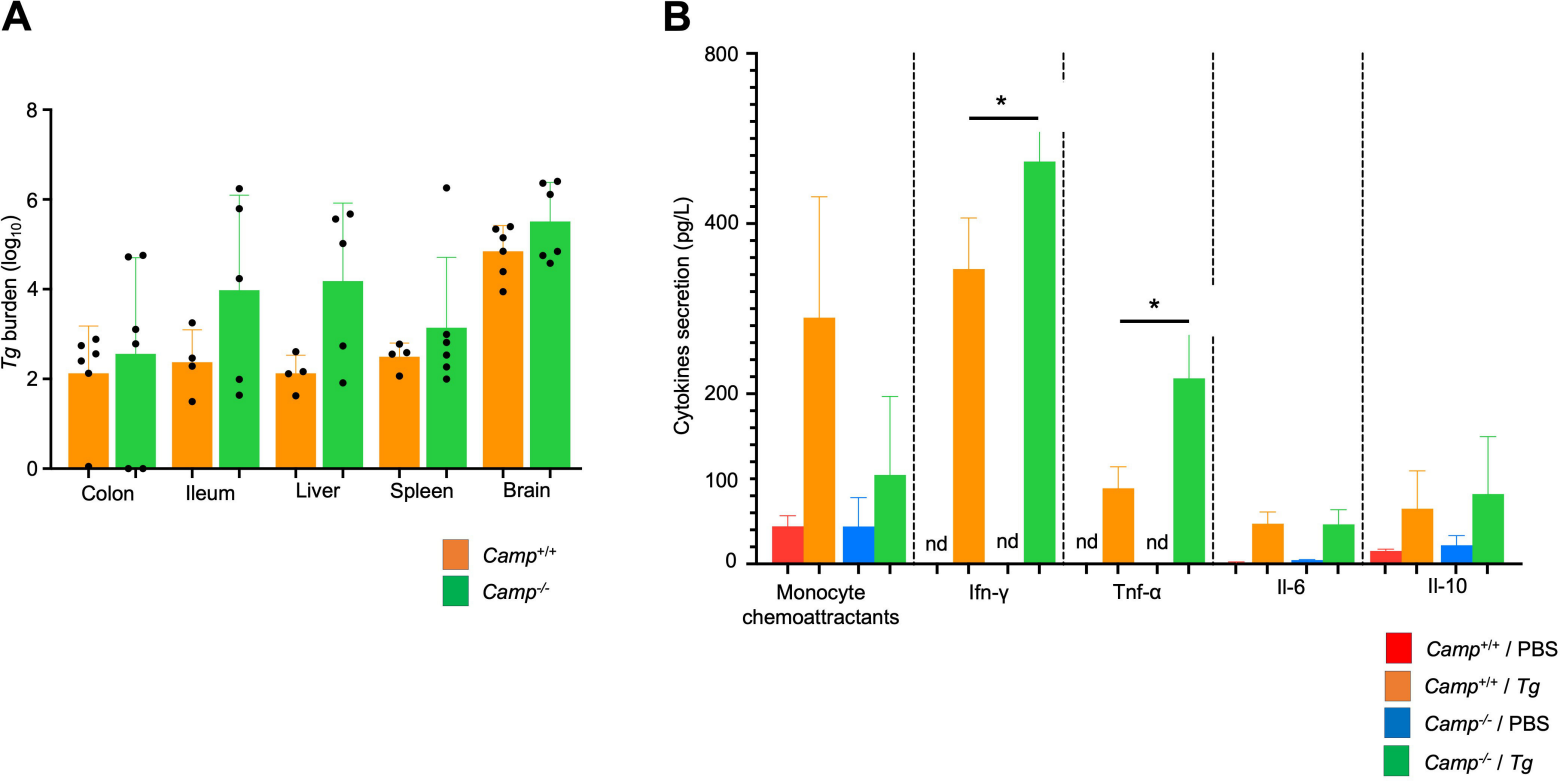
Endogenous cathelicidin prevented systemic pro-inflammatory cytokine production in mice orally infected with *Tg* cysts. *Camp*^+/+^ and *Camp*^-/-^ mice were orally gavaged with five cysts of *Tg* (ME-49, *Tg*) or sham PBS. (**A**) DNA was collected for *Tg* parasite quantification; parasite burdens were not significantly different between *Camp*^+/+^ and *Camp*^-/-^-infected mice. Data are shown as means ± SEM. *P < 0.05 (two-tailed Student’s t-test or one-way ANOVA *post hoc* Tukey’s test for multiple comparisons) was considered significant. (**B**) Synthesis of inflammatory monocyte chemoattractants, Ifn-γ, Tnf-α, Il-6, and Il-10 cytokines in blood plasma of *Camp*^+/+^ and *Camp*^-/-^ *Tg*-infected and sham mice for 14 d, as determined by Luminex. Secretion of Inf-γ and Tnf-α was increased in *Camp*^-/-^-infected mice (*n* = 2–3 mice per group). Data are shown as means ± SEM. *P < 0.05 (two-tailed Student’s t-test) between *Camp*^+/+^ and *Camp*^-/-^ *Tg*-infected mice was considered significant. *Camp^+/+^* and *Camp^-/-^* sham mice are shown only for descriptive purposes and are not statistically analyzed due to the low number of mice per group.

We further examined the systemic impact of this oral toxoplasmosis model to determine whether endogenous cathelicidin could modulate *Tg* infection and mitigate excessive inflammation. The burden of *Tg* tachyzoites in the colon, ileum, liver, spleen, and brain was similar between *Camp*^+/+^ and *Camp^-/-^ Tg*-infected mice, as identified by qPCR targeting B1 genomic repeats of *Tg* (Fig. 8A). However, *Camp^-/-^* mice infected by *Tg* showed increased plasma concentrations of monocyte chemoattractant protein, Ifn-γ, and Tnf-α at 14 days post-challenge (*P* < 0.05) with no differences in the levels of Il-6 and Il-10, compared with *Camp*^+/+^ counterparts (Fig. 8B). Thus, cathelicidin may not inhibit parasite dissemination in the oral toxoplasmosis model but does assist in preventing uncontrollable systemic inflammation associated with the infection.

### Exogenous and endogenous cathelicidin modulated pro-inflammatory TNF-α synthesis and *Tg* killing in macrophages

**Fig 9:**
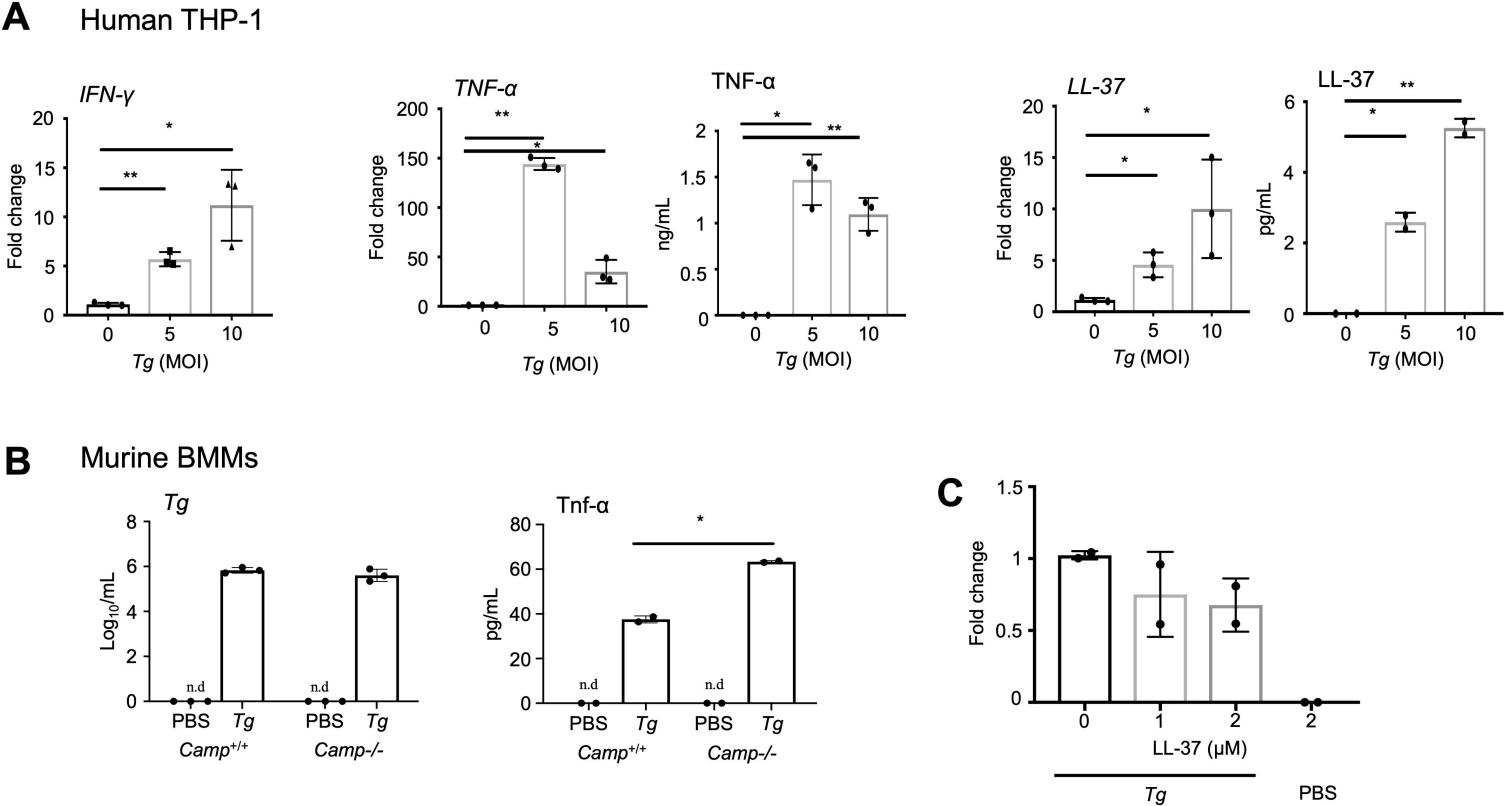
Macrophages upon *Tg* challenge produce pro-inflammatory cytokines and cathelicidins that may exert some microbicidal effect. (**A**) Transcriptional gene expression of human *IFN-γ*, *TNF-α,* and *CAMP* (LL-37) using real-time qRT-PCR, and TNF-α, and LL-37 proteins by commercial ELISAs in human THP-1 macrophages (2 × 10^5^) seeded (12-well plates) and exposed to *Tg* tachyzoites (ME 49, *Tg*) (MOI 0–10, 4 h). Means + SEM are shown. The experiments were run in two or three independent experiments, each performed in triplicate. Each dot represents one independent experimental data point. *P < 0.05 and **P < 0.001 (two-tailed Student’s t-test or one-way ANOVA *post hoc* Tukey’s test for multiple comparisons) were considered significant. (**B**) Intracellular *Tg* burden quantification and secretion of murine Tnf-α using commercial ELISA in BMDMs from *Camp*^+/+^ and *Camp*^-/-^ mice seeded (12-well plate) and exposed to *Tg* tachyzoites (1 × 10^5^ ME 49 *Tg*, *Tg*) for 1 d. Means + SEM are shown. The experiments were run in two or three independent experiments, each performed in triplicate. Each dot represents one independent experimental data point. *P < 0.05 and **P < 0.001 (two-tailed Student’s t-test or one-way ANOVA *post hoc* Tukey’s test for multiple comparisons) were considered significant. (**C**) THP-1 macrophages (2 × 10^5^) were seeded (12-well plates) and treated with recombinant LL-37 (1-2 μM) before being challenged by *Tg* (ME 49, B1 *Tg*) for 4 h. Macrophages were collected to quantify *Tg* using real-time qRT-PCR and relative to the housekeeping gene hGAPDH. Means + SEM are shown (*n* = three independent experiments done in triplicate).

To explore the mechanisms by which cathelicidin enhances host defenses, we focused on macrophages and the synthesis of IFN-γ and TNF-α, which are essential effectors for controlling *Tg* (34). We first demonstrated that human macrophages (dTHP-1) increased the transcriptional gene expression of *IFN-γ* and *TNF-α* and *TNF-α* secretion, as well as endogenous *CAMP* (*LL-37*) expression and secretion in response to *Tg* in a MOI-dependent fashion (*P* < 0.05) (4 h post-challenge; Fig. 9A). Next, we investigated whether endogenous cathelicidin regulates the immunomodulatory function in macrophages. BMDMs from *Camp*^+/+^ and *Camp*^-/-^ mice challenged with *Tg* (ME 49, 1 × 10^5^ for 1 day) showed similar *Tg* burdens (*P* > 0.05, Fig. 9B). However, the secretion of Tnf-α was higher (*P* < 0.05) in *Camp*^-/-^ BMMs infected with *Tg* compared to *Camp*^+/+^ BMMs (Fig. 9B). No secretion of Ifn-γ was detected in BMMs infected with *Tg*. Pre-treatment with recombinant human cathelicidin LL-37 (up to 2 µM) in dTHP-1 cells (1 h) before *Tg* challenge did not reduce *Tg* burden after 4 h (*P* > 0.05) (Fig. 9C). Thus, cathelicidin exhibited unique effects on altering macrophages during *Tg* infection; LL-37 had no direct killing activity against *Tg* but, endogenous cathelicidin contributed to reducing Ifn-γ and Tnf-α secretion.

## DISCUSSION

This study identified the attributes of cathelicidin that prevent the host from experiencing severe weight loss and from succumbing to toxoplasmosis. Along with the roles of cathelicidin as a microbicidal (35, 36), antiparasitic (18), and immunomodulator (37, 38), we demonstrated that cathelicidin limits exacerbated hepatitis in an oral murine model of toxoplasmosis, which mimics the natural route of infection. Our finding that nearly 100% of *Camp*^+/+^-infected mice survived post-challenge, whereas *Camp*^-/-^ mice were terminated 12–14 days post-challenge due to systemic deterioration and humane endpoints, denotes that endogenous cathelicidin is crucial for sustaining the host and preventing overwhelming systemic inflammation. We tested a lesser motile and pathogenic *Tg*, ME 49, a Type ΙΙ (intermediate virulence). *Tg* Type II are frequently associated with human toxoplasmosis (39–41). Parasite-induced pathology and dissemination provoked by Type II and III (lowest virulence, e.g., VEG) (42) can be limited by systemic inflammation (43). However, a more aggressive *Tg* strain, such as Type I (highest virulence, e.g., RH, GT1), usually lethal for mice (44), could overcome these cathelicidin protection mechanisms.

Toxoplasmosis becomes a life-threatening opportunistic infection in immunosuppressed recipients with afflicted visceral organs, including the liver (45). Immunosuppressed patients with toxoplasmosis often experience chronic hepatitis with fatty alteration, fibrosis, and scattered necrotic foci (46–48). There was also an increase in hepatic enzymes in circulation during toxoplasmosis, namely aspartate aminotransferase, alanine aminotransferase, and alkaline phosphatase (ALP) (32). *Tg* infection compromises hepatic function by directly affecting hepatocytes and by enhancing inflammatory responses, leading to exaggerated synthesis of IFN-γ, TNF-α, and IL-12 (49). We revealed severe necrotic hepatitis in mice deficient in cathelicidin infected with *Tg* that likely contributed to mortality. The lack of endogenous cathelicidin promoted waves of pro-inflammatory cytokines, including *Ifn-γ*. Such robust IFN-γ production, likely produced by CD4^+^ and CD8^+^ T cells (50), limits *Tg* replication but promotes tissue damage (51). Although free tachyzoites can appear in the liver during the acute phase of toxoplasmosis (48, 52), we did not detect *Tg* antigenic components in *Camp*^-/-^ livers, suggesting an aberrant hepatitis not correlated with the presence of the parasite.

The high susceptibility and necrotic inflammation in *Camp*^-/-^ livers during *Tg* infection were correlated with higher synthesis of PARP3 and GBPs. PARP3 is a catalytically active enzyme critical for DNA repair, inflammation, and cell death, modifying target proteins and DNA to facilitate post-translational ADP-ribosylation (53). Some PARPs, such as PARP1, exhibit proinflammatory effects on Kupffer cells in fatty liver (54) and promote the release of proinflammatory cytokines via the NF-κB pathway (53). Thus, increased PARP3 expression in *Camp*^-/-^ *Tg*-infected livers may contribute to the exaggerated inflammation and systemic inflammatory cytokine storms. GBPs are a family of IFN-inducible GTPases that contribute to the defense against intracellular pathogens by activating the inflammasome machinery, promoting pyrogenic cytokine production, and inducing pyroptosis (55). Localized within the parasitophorous vacuole in macrophages, GBPs are effectors in immunity against *Tg* (56). Mice lacking a fragment of chromosome 3, which encodes GBPs (1, 2, 3, 5, and 7), are highly susceptible to *Tg* infection, even after IFN-γ stimulation. In the absence of cathelicidin, GBPs may be upregulated to control infection by targeting *Tg-*containing vacuoles.

Following intestinal invasion, *Tg* disseminates throughout the body and crosses the blood-brain barrier, establishing chronic infection with cyst formation in the CNS, primarily in the brain (57). We observed a higher number of *Tg* cysts in the brains of cathelicidin-deficient mice, manifesting an inability to limit parasite persistence and replication in the CNS. No more severe splenitis or grade of colitis or ileitis was reported in *Camp*^+/+^ or *Camp*^-/-^ mice for up to 14 days post-challenge. In contrast, mice infected with a higher number of cysts (>5 cysts) of Type II *Tg* (ME 49 or PRU strain) exhibited ileitis characterized by mucosal epithelial disruption, crypt abscesses, and necrosis of intestinal villi (21–23). The lack of intestinal inflammation in this study could be attributed to a lower dose of *Tg* cysts (*n* = 5) or to the possibility that enteritis had occurred earlier and been resolved by the time of tissue collection. As an observation, the number of goblet cells in the small intestine was reduced in *Camp*^-/-^-infected mice. Cathelicidin stimulates colonic mucus synthesis by up-regulating MUC1 and MUC2 expression (58), and cathelicidin appears to promote mucin secretion (59, 60). Whether the mucin secretagogue role of cathelicidin protects the gut from *Tg* colonization remains elusive. Interestingly, low non-microbicidal amounts of human cathelicidin (LL-37) provoke a rapid and transient increase in epithelial cell permeability by disrupting the TJ proteins occludin and claudin-2 in cultured colonic epithelial cells (61). These temporary increases in permeability could allow *Tg* to enter the intestinal lumen. However, the lack of a susceptible phenotype in wild-type mice indicates that other compensatory mechanisms regulate gut permeability.

While cathelicidin is not primarily produced by hepatocytes (62), leukocytes that produce the peptide influence liver function and disease progression through interactions with liver cells. We showed that cathelicidin from macrophages is increased in response to *Tg,* and that endogenous CRAMP limits TNF-α synthesis. Cathelicidin seems to be a critical defense against intracellular pathogens. Exogenous LL-37 and endogenous LL-37 stimulation reduced intracellular survival of *Mycobacterium tuberculosis* in infected macrophages (36) and *M. paratuberculosis* (63) and promoted the co-localization of mycobacteria-containing phagosomes with lysosomes (64). Synthetic cathelicidins also enhance reactive oxygen species activity via P2X7 receptors in macrophages (65). Cathelicidin can bind to CpG-motif DNA during intracellular infection and deliver bound pathogens to endo-lysosomes for degradation (66). In this regard, CpG responses regulated by cathelicidin may be crucial in toxoplasmosis. CpG-DNA is mainly present in the microbial genome of *Tg* (67), and CpG-DNA of *Tg* upregulates TLR-9 in colonic (Caco-2) epithelial cells (68). Cathelicidins may assist *Tg* in recognizing CpG (perhaps via TLR-9) and promote phagolysosome formation in macrophages. Interactions between IFN-γ and cathelicidin in macrophage biology have not been well characterized. However, IFN-γ-neutralizing antibodies decreased macrophage cathelicidin expression (69).

On the other hand, we found no direct anti-*Tg* effect of endogenous cathelicidins, with no differences in the burden of *Tg* tachyzoites or oocysts in the liver, spleen, or cultured macrophages. This finding contrasts with earlier *in vitro* studies, in which equine cathelicidin (eCATH1; 2 mg/mL) inhibited the survival of bloodstream forms of trypomastigotes from related protozoa (*Trypanosoma brucei brucei*, *Trypanosoma evansi,* and *Trypanosoma equiperdum*) by altering the parasite’s membrane structure (70). The relevance of cathelicidin’s antiprotozoal effects in the host remains elusive, as does the extent to which they confound immunomodulatory roles that limit microbial replication. Thus, cathelicidin could act as a sentinel molecule protecting organs by preventing the overwhelming generalized burden of inflammatory cytokines in response to *Tg*. This finding aligns with studies that demonstrate LL-37 mediates the release of IL-1β and IL-18 in airway epithelial cells infected with *Pseudomonas aeruginosa* (71). Likewise, LL-37 and CRAMP synergized with *Salmonella typhimurium* or *Citrobacter rodentium* to regulate the synthesis of chemokine CXCL8/CXCL1 in the colonic epithelium (72). The role of endogenous cathelicidin in maintaining host well-being may depend on the peptide’s physiological concentrations. In the skin, keratinocytes express limited amounts of cathelicidin in homeostasis (73, 74), but upon bacterial infection or physical injury, cathelicidin concentrations increase and lead to other inflammatory disorders such as psoriasis (75) and rosacea (76).

In summary, this study provides compelling evidence that cathelicidin functions predominantly as an immunomodulatory molecule rather than as a direct effector against *Tg*. Its role is especially critical in the infected liver, where cathelicidin effectively restrains pathological inflammation while exhibiting minimal direct antiparasitic activity. These findings support a model in which cathelicidin acts as a regulatory “shield,” preserving host tissue integrity and survival by preventing immune-mediated damage during toxoplasmosis. Although the study is limited by the number of animals analyzed and the use of a single *Tg* strain, these constraints do not diminish the central conclusion and instead underscore the importance of further investigations to fully elucidate cathelicidin biology and its protective function in host–parasite interactions.

## Data Availability

The proteomic raw data are available in PRIDE under accession no. PXD062489.

## AFTER EPUB

*[This article was published on 23 February 2026 with an error in the author byline. The byline was corrected in the current version, posted on 23 March 2026.]*

## SUPPLEMENTAL MATERIAL

Table S1 iai.00708-25-s0001.docx Differentially expressed genes in the liver of Camp^+/+^ versus Camp^-/-^ mice challenged with *Toxoplasma gondii*.

Table S2 iai.00708-25-s0002.xlsx Liver proteomic data.

ASM does not own the copyrights to Supplemental Material that may be linked to, or accessed through, an article. The authors have granted ASM a non-exclusive, world-wide license to publish the Supplemental Material files. Please contact the corresponding author directly for reuse.
