# Supplementary material for: Endogenous cathelicidin protects against Toxoplasma gondii-associated liver damage
Source: Infect Immun. 2026 Feb 23;94(5):e00708-25. doi: 10.1128/iai.00708-25 (PMC13163205; doi:10.1128/iai.00708-25)
Supplement: Table S1 — Differentially expressed genes in the liver of Camp+/+ versus Camp-/- mice challenged with Toxoplasma gondii. [file iai.00708-25-s0001.docx]

**Table S1.** Differentially expressed genes in the liver of *Camp^+/+^* versus *Camp^-/-^* mice challenged with *Toxoplasma gondii*.

| *Gene* | Protein Name | log_2_FC | Adjusted p value |
| --- | --- | --- | --- |
| *Myo1f* | Myosin-1F-like protein | 12.67 | 0.000654938 |
| *Nrbp1* | Nuclear receptor-binding protein | 11.79 | 0.0013875 |
| *Iglc2;Iglc3* | Ig lambda-2 chain C region | 5.64 | 0.00452025 |
| *NaNgs* | N-acetylglutamate synthase, mitochondrial | 1.33 | 2.079E-07 |
| *Gls2* | Glutaminase liver isoform, mitochondrial | 0.99 | 0.00121625 |
| *Gldc* | Glycine dehydrogenase (decarboxylating), mitochondrial | 0.81 | 0.00452025 |
| *Aldh1b1* | Aldehyde dehydrogenase X, mitochondrial | 0.76 | 0.002749688 |
| *Alas1* | 5-aminolevulinate synthase, nonspecific, mitochondrial | 0.59 | 0.025240385 |
| *Acy1* | Aminoacylase-1 | -0.74 | 0.00121625 |
| *Aldh18a1* | Delta-1-pyrroline-5-carboxylate synthase | -1.12 | 0.037676297 |
| *Parp3* | Protein mono-ADP-ribosyltransferase PARP3 | -1.32 | 2.25488E-06 |
| *Gbp2b* | Guanylate binding protein 1 | -5.19 | 0.0002905 |
